# The Ubiquitin Conjugating Enzyme UbcD1 is Required for Notch Signaling Activation During *Drosophila* Wing Development

**DOI:** 10.3389/fgene.2021.770853

**Published:** 2021-10-12

**Authors:** Fengchao Zhang, Yao Chen, Jie Shen, Junzheng Zhang

**Affiliations:** MOA Key Lab of Pest Monitoring and Green Management, College of Plant Protection, China Agricultural University, Beijing, China

**Keywords:** ubiquitin conjugating enzyme, UbcD1, Notch, *Drosophila*, wing

## Abstract

Notch signaling pathway plays crucial roles in animal development. Protein ubiquitination contributes to Notch signaling regulation by governing the stability and activity of major signaling components. Studies in *Drosophila* have identified multiple ubiquitin ligases and deubiquitinating enzymes that modify Notch ligand and receptor proteins. The fate of ubiquitinated substrates depend on topologies of the attached ubiquitin chains, which are determined by the ubiquitin conjugating enzymes (E2 enzymes). However, which E2 enzymes participate in Notch signal transduction remain elusive. Here, we report that the E2 enzyme UbcD1 is required for Notch signaling activation during *Drosophila* wing development. Mutations of *UbcD1* lead to marginal nicks in the adult wing and reduction of Notch signaling targets expression in the wing imaginal disc. Genetic analysis reveal that UbcD1 functions in the signaling receiving cells prior to cleavage of the Notch protein. We provide further evidence suggesting that UbcD1 is likely involved in endocytic trafficking of Notch protein. Our results demonstrate that UbcD1 positively regulates Notch signaling and thus reveal a novel role of UbcD1 in development.

## Introduction

Notch signaling pathway plays crucial roles in developmental processes such as tissue patterning, cell proliferation and cell fate determination (Bray, 2016). Malfunction of Notch signaling results in various malignant diseases in human, including neuropsychiatric diseases, metabolic disorders and multiple types of cancer (Salazar and Yamamoto, 2018). The core components and signal transduction routes of Notch signaling are highly conserved among the animal kingdom (Fortini, 2009). Named after the wing margin nicking phenotype observed in the *Drosophila* mutant, the *Notch* gene encodes a transmembrane protein which functions as signal receptor (Bray, 2016). Binding of Notch with ligand proteins Delta or Serrate, which are presented at the membrane of signal sending cells leads to a series of proteolytic cleavage of the Notch protein (Fortini, 2009). As a consequence, the Notch intracellular domain (NICD) is released and translocates into nucleus in the signal receiving cells (Kopan and Ilagan, 2009). NICD interacts with the transcription factor Suppressor of Hairless [Su(H)] and the co-activator Mastermind (Mam) to form a ternary complex. The Su(H)/NICD/Mam complex recognizes specific cis-regulatory regions and activates transcription of Notch target genes. In the absence of signal input, Su(H) recruits co-repressors and inhibits the expression of Notch targets (Kopan and Ilagan, 2009).

The Notch signaling is tightly controlled by auxiliary factors that modulate the expression, stability and activity of the core components (Fortini, 2009). Recent studies have revealed that protein ubiquitination is extensively involved in the regulation of Notch signaling pathway (Le Bras et al., 2011; Weinmaster and Fischer 2011). Protein ubiquitination is a reversible post-translational modification catalyzed by four distinct enzymes. The E1 (Ub-activating) and E2 (Ub-conjugating) enzymes are responsible for activating and conjugating the ubiquitin (Ub) moiety, respectively. The E3 (Ub ligases) enzyme recognizes specific substrates and transfers Ub from E2 onto them. The deubiquitinating enzyme (DUB) removes Ub from substrate proteins to counteract the ubiquitination process (Grabbe et al., 2011). E2 enzymes are now considered as the main determinant for the topology of ubiquitin chains, which directs the ubiquitinated substrates towards distinct fates (Ye and Rape, 2009).

Multiple E3s and DUBs have been demonstrated to regulate Notch signaling during fly development (Moretti and Brou, 2013). In the signal sending cells, E3 ligases Neuralized (Neur) and Mind bomb (Mib1) promote mono-ubiquitination of the ligand proteins Delta and Serrate to facilitate their endocytosis (Yeh et al., 2000; Lai et al., 2001; Pavlopoulos et al., 2001; Itoh et al., 2003; Le Borgne and Schweisguth, 2003; Li and Baker, 2004; Lai et al., 2005). Ubiquitination and endocytosis of ligand proteins are required for initiation of signal transduction in various tissues (Yeh et al., 2001; Le Borgne et al., 2005; Pitsouli and Delidakis, 2005; Wang and Struhl, 2005; Skwarek et al., 2007; Miller and Posakony, 2018). In the signal receiving cells, Notch molecules are ubiquitinated by E3 ligases Nedd4 and Suppressor of deltex [Su(dx)] and targeted for lysosomal degradation to avoid ligand independent activation (Cornell et al., 1999; Mazaleyrat et al., 2003; Sakata et al., 2004; Wilkin et al., 2004; Dalton et al., 2011). The E3 ubiquitin ligase Deltex (Dx) was isolated as a positive regulator of Notch signaling which genetically and physically interacts with Notch (Xu and Artavanis-Tsakonas, 1990; Diederich et al., 1994; Matsuno et al., 1995; Matsuno et al., 2002). Subsequent studies reveal that Dx promotes ubiquitination and ligand independent activation of Notch through the endocytic machinery (Hori et al., 2004; Wilkin et al., 2008; Hori et al., 2011; Yamada et al., 2011). Interestingly, Dx is also capable of inhibiting Notch activation in certain developmental contexts (Mukherjee et al., 2005; Fuwa et al., 2006; Dutta et al., 2017). The E3 ligase cbl is found to target both Dl (Wang et al., 2010) and Notch (Bala Tannan et al., 2018) for degradation. The DUB enzyme Fat facets (Faf) enhances Delta endocytosis to promote Notch signaling during fly eye development (Cadavid et al., 2000; Chen and Fischer, 2000; Chen et al., 2002; Overstreet et al., 2004), while another DUB enzyme USP5 negatively regulates Notch signaling in the same tissue (Ling et al., 2017). Several other DUBs have been implicated in Notch signaling regulation during wing development, but their substrates are still elusive (Zhang et al., 2012).

To date, very little is known about the roles of E2 enzymes in Notch signaling. Here we report that the E2 enzyme UbcD1 (also known as effete) positively regulates Notch signaling activity in the signal receiving cells during *Drosophila* wing development. UbcD1 is a highly conserved class I E2 enzyme (Treier et al., 1992), which plays important roles in a broad spectrum of cellular and developmental events. UbcD1 participates in regulation of telomere behavior (Cenci et al., 1997; Cipressa et al., 2013), apoptosis (Ryoo et al., 2002; Yeh and Bratton, 2013), innate immunity (Chen et al., 2017), dendrite pruning (Kuo et al., 2006), oogenesis (Ohlmeyer and Schupbach, 2003; Chen et al., 2009), neuroblast proliferation (Li et al., 2014) as well as Hedgehog (Hh) signaling and fly wing patterning (Pan et al., 2017). Our study represents the first analysis for the role of UbcD1 in Notch signaling pathway, which will help to understand the functional complexity and diversity of UbcD1.

## Materials and Methods

### Fly Stocks

All fly stocks and crosses were maintained at 25°C on standard media. The stocks used in this study are: *FRT82B,UbcD1* ^*s1782*^ */TM6B* (#111415; Kyoto Stock Center); *FRT82B,UbcD1* ^*8*^ */TM6B* (Chen et al., 2009); *FRT82B,UbcD1* ^*mer1*^ */TM6B* (Pan et al., 2017); *NRE-EGFP* (#30728; Bloomington *Drosophila* Stock Center, BDSC); *dpp-Gal4, UAS-mCD8-GFP/TM3*, *dpp-Gal4, UAS-mRFP/TM3*, *C5-Gal4,UAS-GFP/TM6B* and *C96-Gal4,UAS-GFP/TM*6B (Zhang et al., 2012; Li et al., 2019); *UbcD1* RNAi (#26011; Vienna *Drosophila* Resource Center); *UAS-Dl* (#26694; BDSC); *UAS-N* ^*FL*^ (#52309; BDSC); *UAS-NICD* (Xie et al., 2014); *UAS-UbcD1* ^*WT*^ and *UAS-UbcD1* ^*C85A*^ (Pan et al., 2017); *tub-GFP-LAMP1* (Akbar et al., 2009). The *Ubx-Flp*; *FRT82B*, *Ubi-RFP*/*TM6B* and *Ubx-Flp*; *FRT82B*, *Ubi-GFP*/*TM6B* stock were used to induce somatic clones in wing disc. The *hsFlp*; *Tub-Gal4, UAS-GFP*/*Cyo*; *FRT82B*, *Tub-Gal80* stock was used to generate MARCM clones as previously described (Chang et al., 2021).

**FIGURE 1:**
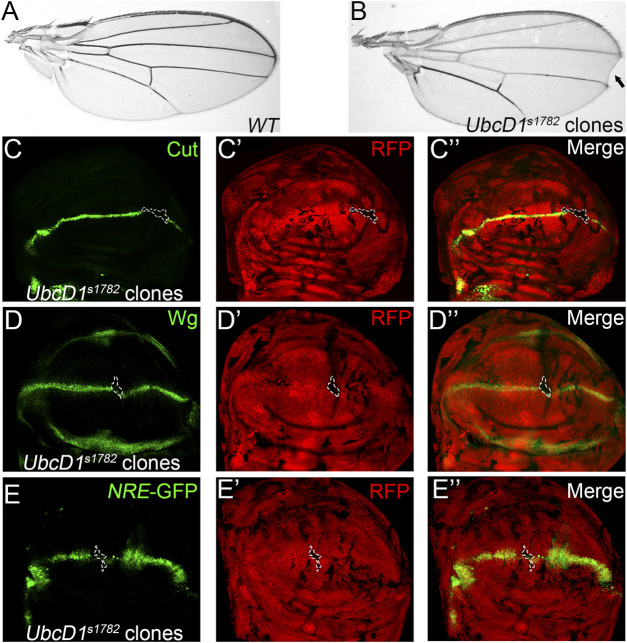
*UbcD1* ^*s1782*^ shows phenotypes that resemble Notch LOF in *Drosophila* wing. **(A–B)** Wing of the parental *Ubx-Flp; FRT82B, Ubi-RFP* stock is shown as wild type control **(A)**. Wing margin loss **(black arrow)** is observed in fly wings bearing *UbcD1* ^*s1782*^ homozygous clones **(B)**. **(C–E)** Expression of Notch signaling targets Cut **(C)**, Wg **(D)** and the reporter *NRE*-GFP **(E)** are abolished in *UbcD1* ^*s1782*^ homozygous mutant clones. Mutant clones are marked by absence of RFP. Representative mutant clones are circled by dashed lines.

**FIGURE 2:**
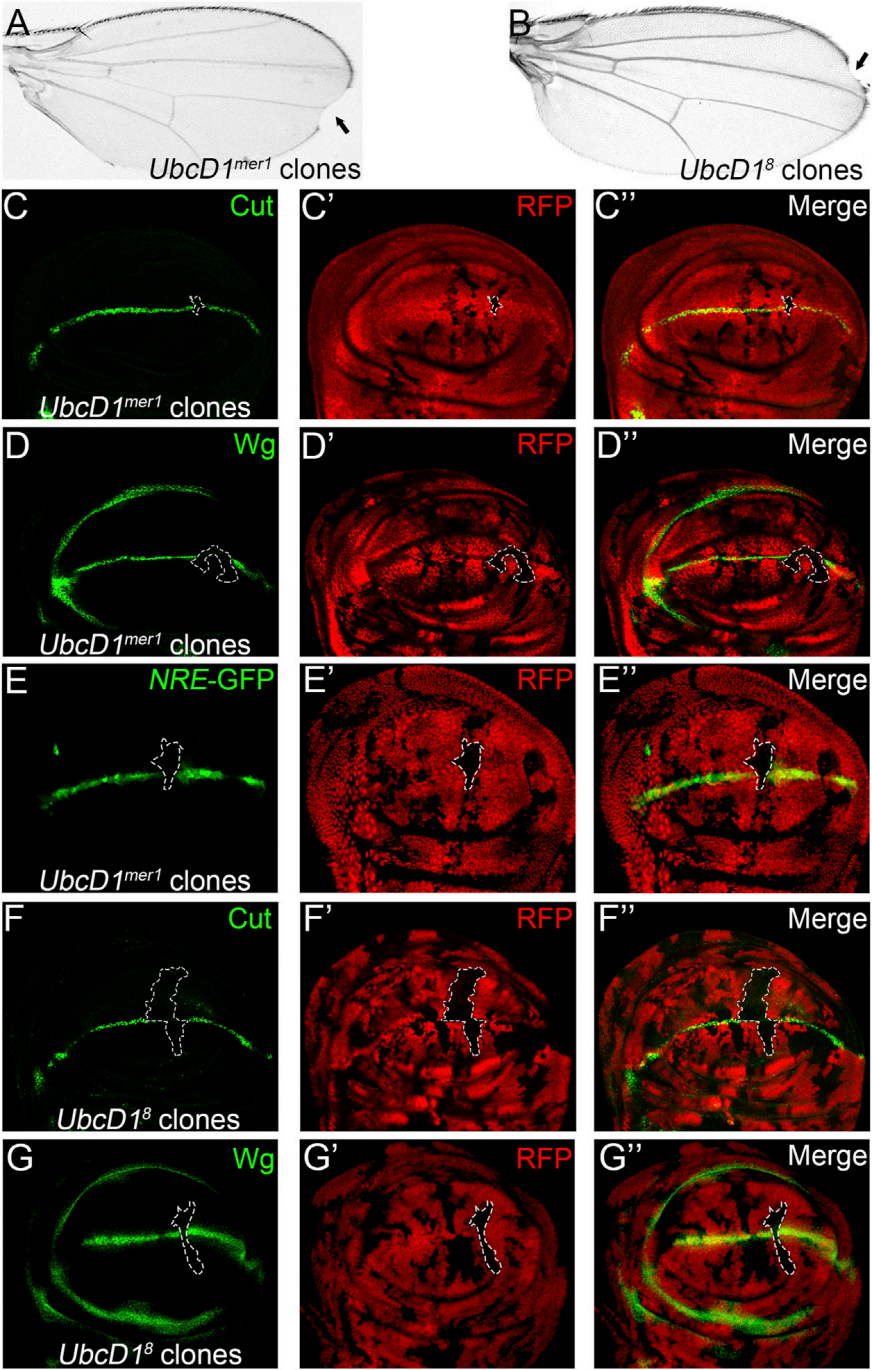
*UbcD1* mutants inhibit Notch signaling activity in *Drosophila* wing. **(A–-B)** Wing margin nicks** (black arrow)** are observed in fly wings bearing *UbcD1* ^*mer1*^ **(A)** and *UbcD1* ^*8*^ **(B)** homozygous clones. **(C–E)** Expression of Notch signaling targets Cut **(C)**, Wg **(D)** and the reporter *NRE*-GFP **(E)** are abolished in *UbcD1* ^*mer1*^ homozygous mutant cells. **(F–G)** Expression of Cut **(F)** and Wg **(G)** are abolished in a subset of *UbcD1* ^*8*^ homozygous cells. Mutant clones are marked by absence of RFP. Representative mutant clones are circled by dashed lines. The expression pattern of Cut, Wg and *NRE*-GFP in wild type wing discs are shown in Supplementary Figure S1.

**FIGURE 3:**
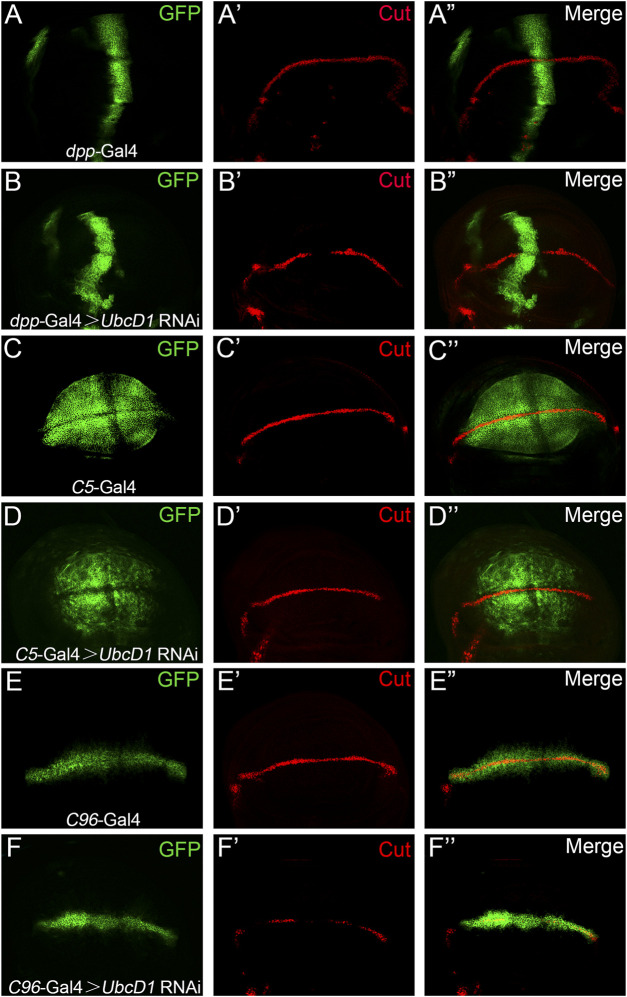
*UbcD1* knock-down in the signaling receiving cells inhibit Notch activity. **(A–B)** When driven by *dpp*-Gal4 in the anterior-posterior border region **(A)**, *UbcD1* RNAi leads to reduction of Cut expression **(B)**. **(C–D)** The *C5*-Gal4 drives GFP expression in the signal-sending cells **(C)**. Expression of Cut are not affected when *UbcD1* RNAi are driven by the *C5*-Gal4 **(D)**. **(E–F)** The *C96*-Gal4 expression domain is restricted within the signal-receiving cells **(E)**. Knocking-down *UbcD1* in the signal-receiving cells by *C96*-Gal4 disrupts Cut expression **(F)**. The Gal4 expression domain are marked by GFP.

**FIGURE 4:**
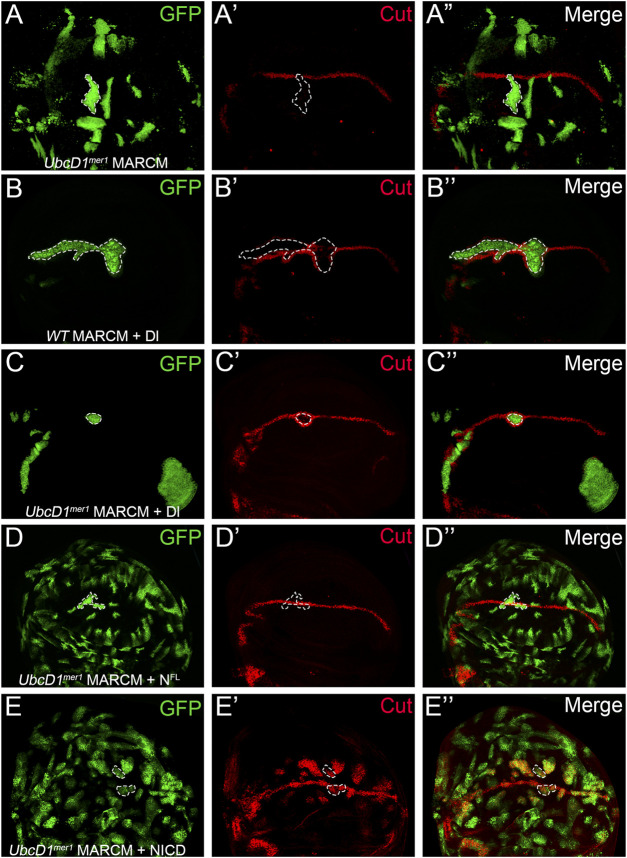
UbcD1 functions upstream of Notch protein processing. **(A)** In *UbcD1* ^*mer1*^ MARCM clones, the expression of Cut are reduced. **(B–C)** Over-expression of Dl in both wild type **(B)** and *UbcD1* ^*mer1*^ mutant cells **(C)** result in induction of Cut in cells surrounding the MARCM clone. **(D)** The full-length Notch protein restores Cut expression in *UbcD1* ^*mer1*^ homozygous cells. **(E)** NICD robustly induces Cut expression in *UbcD1* ^*mer1*^ mutant cells. MARCM clones are marked by GFP. Representative clones are circled by dashed lines.

**FIGURE 5:**
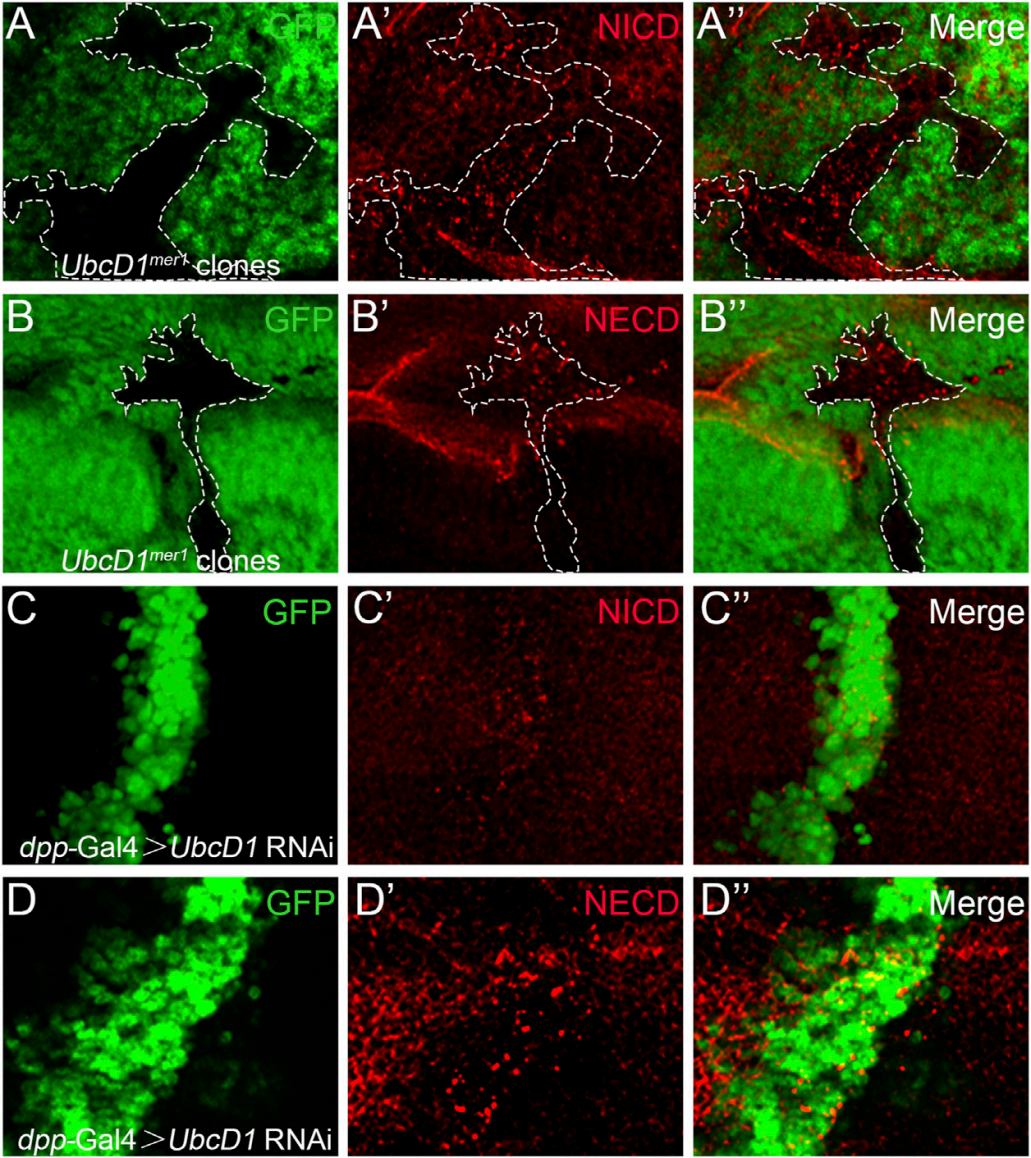
UbcD1 affects Notch distribution. **(A–B)** In *UbcD1* ^*mer1*^ mutant cells, Notch proteins form puncta when labeled by antibodies against NICD **(A)** and NECD **(B)**. Mutant clones are marked by absence of GFP. Representative mutant clones are circled by dashed lines. **(C–D)** In *UbcD1* RNAi cells, Notch proteins form puncta when labeled by antibodies against NICD **(C)** and NECD **(D)**. The RNAi expressing cells are marked by GFP. Panels **(A–D)** are magnification of a portion of Supplementary Figure S2.

**FIGURE 6:**
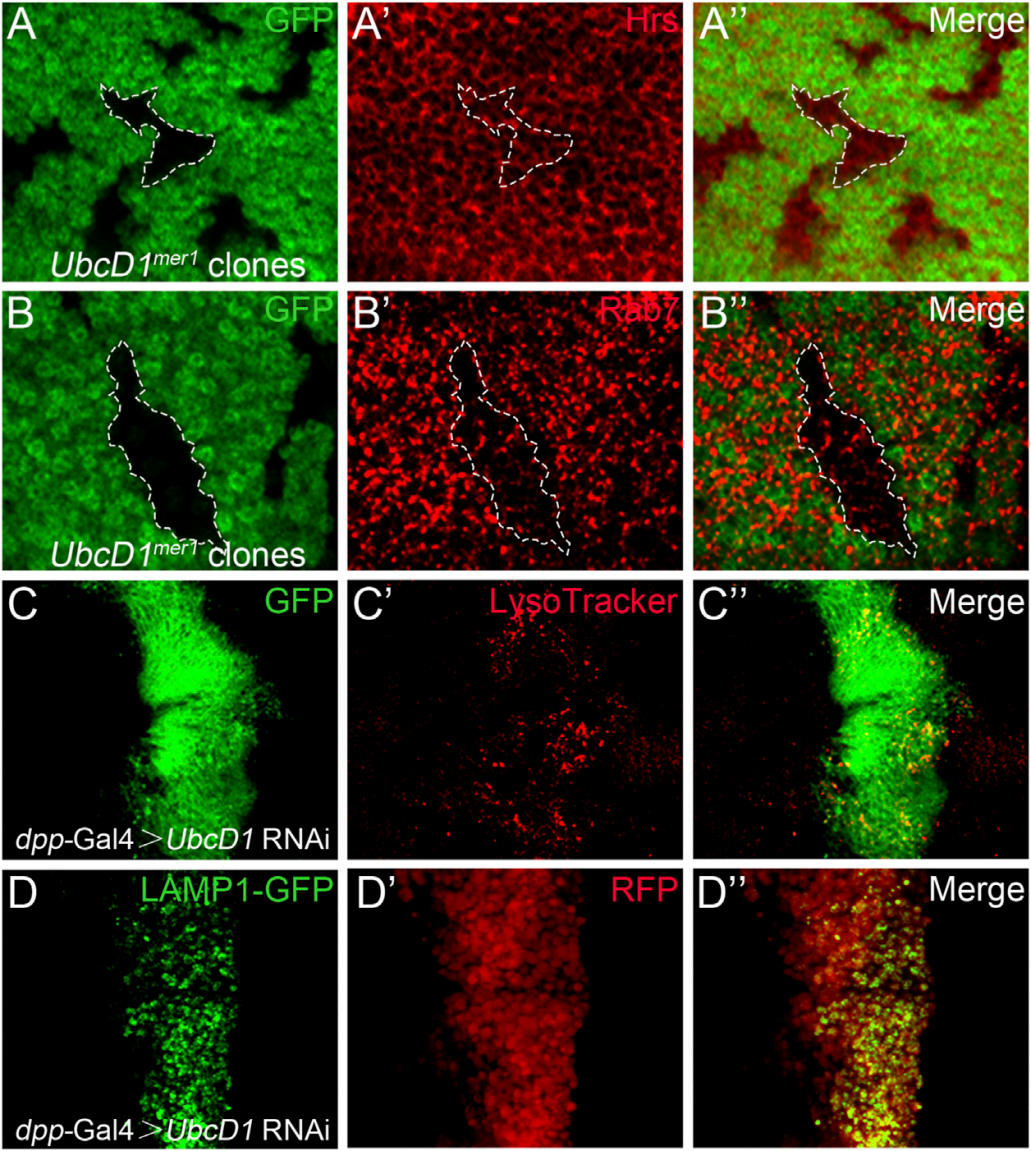
UbcD1 affects endolysosomal machinery. **(A–B)** In *UbcD1* ^*mer1*^ mutant cells, Hrs positive early endosomes are not affected **(A)** while Rab7 association with endosomes are reduced **(B)**. Mutant clones are marked by absence of GFP. Representative mutant clones are circled by dashed lines. **(C–D)** In *UbcD1* RNAi cells, accumulation of LysoTracker **(C)** and GFP-LAMP1 **(D)** are evident. The RNAi expressing cells are marked by GFP **(C)** or RFP **(D)**. Panels **(A–D)** are magnification of a portion of Supplementary Figure S3.

**FIGURE 7:**
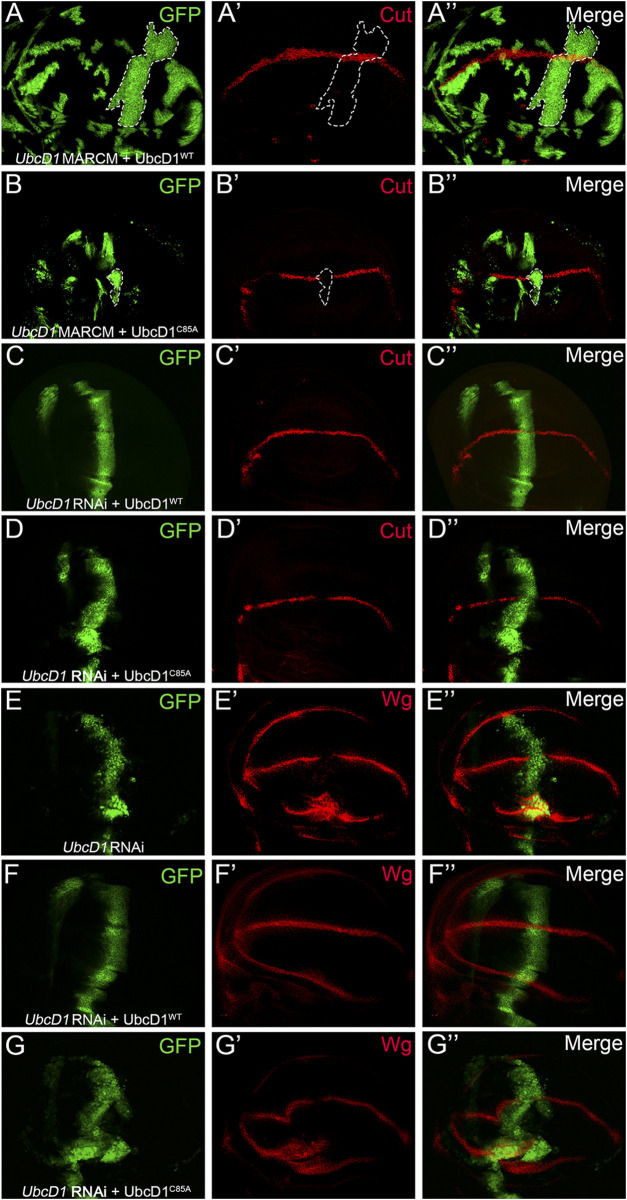
UbcD1 functions as an E2 enzyme. **(A–B)** In *UbcD1* ^*mer1*^ mutant cells, UbcD1^WT^ **(A)** but not UbcD1^C85A^ **(B)** is sufficient to restore the expression of Cut. MARCM clones are marked by GFP. Representative clones are circled by dashed lines. **(C–D)** UbcD1^WT^ **(C)** but not UbcD1^C85A^ **(D)** is able to rescue the reduction of Cut expression caused by UbcD1 RNAi. **(E–G)** RNAi knock-down of *UbcD1* leads to inhibition of Wg expression **(E)**, which is rescued by UbcD1^WT^ **(F)** but not UbcD1^C85A^ **(G)**. Note that Wg are accumulated in cells at the edge of wing pouch upon *UbcD1* RNAi **(E, G)**. The RNAi expressing cells are marked by GFP.

The genotypes in the experiments are listed below:

Figure 1A: *Ubx-Flp; FRT82B, Ubi-RFP*.
Figure 1B–D: *FRT82B, UbcD1* ^*s1782*^ *× Ubx-Flp; FRT82B, Ubi-RFP*.
Figure 1E: *NRE-GFP; FRT82B, UbcD1* ^*s1782*^ × *Ubx-Flp; FRT82B, Ubi-RFP*.
Figure 2A, C, D: *FRT82B, UbcD1* ^*mer1*^ *× Ubx-Flp; FRT82B, Ubi-RFP*.
Figure 2B, F, G: *FRT82B, UbcD1* ^*8*^ *× Ubx-Flp; FRT82B, Ubi-RFP*.
Figure 2E: *NRE-GFP; FRT82B, UbcD1* ^*mer1*^ × *Ubx-Flp; FRT82B, Ubi-RFP*.
Figure 3A: *dpp-Gal4, UAS-mCD8-GFP*.
Figure 3B: *dpp-Gal4, UAS-mCD8-GFP* × *UbcD1* RNAi.
Figure 3C: *C5-Gal4, UAS-GFP*.
Figure 3D: *C5-Gal4, UAS-GFP* × *UbcD1* RNAi.
Figure 3E: *C96-Gal4, UAS-GFP*.
Figure 3F: *C96-Gal4, UAS-GFP × UbcD1* RNAi.
Figure 4A: *hsFlp; Tub-Gal4, UAS-GFP; FRT82B, Tub-Gal80* × *FRT82B, UbcD1* ^*mer1*^.
Figure 4B: *hsFlp; Tub-Gal4, UAS-GFP; FRT82B, Tub-Gal80* × *UAS-Dl; FRT82B*.
Figure 4C: *hsFlp; Tub-Gal4, UAS-GFP; FRT82B, Tub-Gal80* × *UAS-Dl; FRT82B, UbcD1* ^*mer1*^.
Figure 4D: *hsFlp; Tub-Gal4, UAS-GFP; FRT82B, Tub-Gal80* × *UAS-N* ^*FL*^ *; FRT82B, UbcD1* ^*mer1*^.
Figure 4E: *hsFlp; Tub-Gal4, UAS-GFP; FRT82B, Tub-Gal80* × *UAS-NICD; FRT82B, UbcD1* ^*mer1*^.
Figure 5A, B: *FRT82B, UbcD1* ^*mer1*^ *× Ubx-Flp; FRT82B, Ubi-GFP*.
Figure 5C, D: *dpp-Gal4, UAS-mCD8-GFP* × *UbcD1* RNAi.
Figure 6A, B: *FRT82B, UbcD1* ^*mer1*^ *× Ubx-Flp; FRT82B, Ubi-GFP*.
Figure 6C: *dpp-Gal4, UAS-mCD8-GFP* × *UbcD1* RNAi.
Figure 6D: *dpp-Gal4, UAS-mRFP* × *tub-GFP-LAMP1; UbcD1* RNAi.
Figure 7A: *hsFlp; Tub-Gal4, UAS-GFP; FRT82B, Tub-Gal80* × *UAS-UbcD1* ^*WT*^ *; FRT82B, UbcD1* ^*mer1*^.
Figure 7B: *hsFlp; Tub-Gal4, UAS-GFP; FRT82B, Tub-Gal80* × *UAS-UbcD1* ^*C85A*^ *; FRT82B, UbcD1* ^*mer1*^.
Figure 7C, F: *dpp-Gal4, UAS-mCD8-GFP* × *UAS-UbcD1* ^*WT*^ *; UbcD1* RNAi.
Figure 7D, G: *dpp-Gal4, UAS-mCD8-GFP* × *UAS- UbcD1* ^*C85A*^ *; UbcD1* RNAi.
Figure 7E: *dpp-Gal4, UAS-mCD8-GFP* × *UbcD1* RNAi.

### Immunostaining and Microscopy

Third-instar larvae were dissected in cold PBS and fixed with 4% paraformaldehyde for 15 min at room temperature. The wing discs were washed with 0.1% Triton X-100 in PBS (PBST) and blocked in 0.2% BSA in PBST for 1 h before incubating with primary antibodies overnight at 4°C. The primary antibodies used in this study are: mouse anti-Cut (1:200; 2B10; Developmental Studies Hybridoma Bank, DSHB), mouse anti-Wg (1:200; 4D4; DSHB), mouse anti-NICD (1:200; C17.9C6; DSHB), mouse anti-NECD (1:200; C458.2H; DSHB), mouse anti-Dl (1:200; C594.9B; DSHB), mouse anti-Rab7 (1:200; Rab7; DSHB), mouse anti-Hrs (1:200; Hrs8-2; DSHB). After washing with PBST, wing discs were immersed in second antibodies conjugated with Alexa Fluor 488 (1:200; Invitrogen) or Alexa Fluor 568 (1:200; Invitrogen) for 1 h at room temperature. After washing with PBST for three times, wing discs were dissected and mounted in the VECTASHIELD mounting medium (Vector Laboratories). For LysoTracker staining, wing discs were dissected in Schneider’s *Drosophila* medium (#21720024, Thermo Fisher) and incubated in medium containing LysoTracker (1:20000; L7528; Invitrogen) for 5 min at room temperature. After washed by fresh medium, the wing discs were mounted and imaged. The fluorescence images were acquired with Leica SP8 confocal microscope and assembled in Photoshop and ImageJ.

Adult wings were dissected from flies after fixed in isopropanol for at least 24 h and mounted in 50% glycerol. Images of adult wings were captured using a Leica DMIL inverted microscope equipped with a QImaging QICAM Fast 1394 digital camera.

## Results

### UbcD1 Regulates Notch Signaling in the *Drosophila* Wing

Using a somatic mosaic screen strategy (Ren et al., 2018), we isolated an *UbcD1* allele that impairs Notch signaling during fly wing development. Marginal nicks were observed in fly wings bearing homozygous *UbcD1* ^*s1782*^ clones (Figures 1A,B), a typical phenotype caused by Notch loss-of-function (LOF) (Blair, 2007; Bray, 2016). Notch activates the expression of target genes such as *cut* and *wingless* (*wg*) in cells located at the dorsal-ventral (D/V) boundary in the wing imaginal disc (Supplementary Figures S1A,B). The expression of Cut and Wg were abolished in *UbcD1* ^*s1782*^ homozygous cells (Figures 1C,D). The transcriptional activity of Notch signaling could be visualized by the *NRE*-GFP reporter (Saj et al., 2010), and the expression of *NRE*-GFP was also dampened in *UbcD1* ^*s1782*^ homozygous clones (Figure 1E and Supplementary Figure S1C). These observations suggest that Notch signaling activity is disrupted in *UbcD1* ^*s1782*^ mutant wing disc cells.

To further establish a role of UbcD1 in Notch signaling transduction, two additional *UbcD1* alleles were tested. Both *UbcD1* ^*mer1*^ and *UbcD1* ^*8*^ are LOF alleles that have been shown to cause developmental defects in various fly tissues (Chen et al., 2009; Pan et al., 2017). Upon induction of somatic mosaic clones, both alleles led to wing margin nicks (Figures 2A,B). Expression of Cut (Figure 2C), Wg (Figure 2D) as well as the *NRE*-GFP reporter (Figure 2E) were reduced in *UbcD1* ^*mer1*^ homozygous cells. Similarly, *UbcD1* ^*8*^ mutant cells were also deficient of Cut (Figure 2F) and Wg (Figure 2G) expression. Taken together, we conclude that UbcD1 positively regulates Notch signaling during fly wing development.

### UbcD1 Functions in the Signal Receiving Cells

Notch signaling operates among two group of cells, UbcD1 might function in either signal sending or receiving cells in the process of signal transduction. Cell type specific RNAi experiments were performed to further distinguish in which group of cells Ubcd1 are required. Knock-down of *UbcD1* by a transgenic RNAi construct resulted in significant inhibition of Cut expression in the wing disc (Figures 3A,B). The *C5*-Gal4 (Hall et al., 2017; Li et al., 2019) was used to drive *UbcD1* RNAi in the signal-sending cells. Knock-down of *UbcD1* in the signal sending cells showed little impact on Cut expression (Figures 3C,D). When the *C96*-Gal4 was used to drive *UbcD1* RNAi in the signal-receiving cells (Zhang et al., 2012), reduction of Cut was observed (Figures 3E,F). Therefore, UbcD1 likely functions in the signal-receiving cells to regulate Notch signaling activity.

To dissect how UbcD1 regulates Notch signal transduction, we used the MARCM system (Lee and Luo, 2001) to overexpress Dl and Notch proteins in *UbcD1* ^*mer1*^ mutant cells. In *UbcD1* ^*mer1*^ MARCM clones which are positively marked by GFP, the expression of Cut was abolished (Figure 4A). Expression of Dl in wild type cells led to induction of Cut in cells surrounding the MARCM clones (Figure 4B), as they received excessive signal inputs from cells inside the clone. In *UbcD1* ^*mer1*^ mutant cells, overexpression of Dl was still capable of inducing Cut expression in the surrounding cells (Figure 4C). These results confirm that UbcD1 is dispensable in the signal sending cells.

In clones located at the D/V boundary, over-expression of Dl was insufficient to rescue Cut expression (Figure 4C). In contrast, the full-length Notch protein was able to restore the expression of Cut in *UbcD1* ^*mer1*^ homozygous cells (Figure 4D). When NICD was introduced into *UbcD1* ^*mer1*^ mutant cells, ectopic expression of Cut was robustly induced (Figure 4E). These genetics analysis suggests that UbcD1 functions in the signal receiving cells, presumably at early steps before the cleavage of full-length Notch protein.

### UbcD1 Affects Notch Protein Distribution

Giving that UbcD1 functions up-stream of Notch protein processing, the potential effects on Notch protein were further examined. In *UbcD1* ^*mer1*^ homozygous mutant cells, Notch proteins accumulated as puncta when labeled by an antibody recognizing the intracellular domain (Figure 5A and Supplementary Figure S2A). Similar distribution defect was observed using a second antibody raised against the extracellular domain of Notch protein (Figure 5B and Supplementary Figure S2B). Consistently, RNAi knock-down of *UbcD1* also resulted in aggregation of Notch proteins (Figures 5C,D and Supplementary Figures S2C,D).

Accumulation of Notch proteins accompanied with reduction of signaling activity have been found in mutations of the endolysosomal pathway components (Vaccari et al., 2008; Vaccari et al., 2010; Ren et al., 2018). Therefore, whether UbcD1 is involved in the endolysosomal machinery was investigated. In *UbcD1* ^*mer1*^ mutant cells, early endosomes as labeled by Hrs were not significantly affected (Figure 6A and Supplementary Figure S3A), but formation of Rab7-positive late endosomes was inhibited (Figure 6B and Supplementary Figure S3B). Lacking of Rab7 associated late endosomes might disrupt subsequent events such as endolysosome acidification and cargo degradation. Interestingly, when applied to live wing discs, strong accumulation of the acidotrophic fluorescent dye LysoTracker was observed in *UbcD1* RNAi cells (Figure 6C and Supplementary Figure S3C). This result indicates that despite the reduction of late endosome maturation, acidification of endocytic organelles are enhanced in *UbcD1* RNAi cells. We further examined the lysosomal activity using a GFP-Lamp1 fusion protein that undergoes rapid lysosomal degradation in physiological context (Akbar et al., 2009). GFP-Lamp1 was hardly detectable in wild type wing imaginal disc cells, while knock-down of *UbcD1* by RNAi caused a significant accumulation of GFP-Lamp1 (Figure 6D and Supplementary Figure S3D). These results indicate that UbcD1 might safeguard the integrity of the endolysosomal machinery to promote Notch signal transduction.

### UbcD1 Regulates Notch Signaling as an E2 Enzyme

A conserved Cystine residue at position 85 (C85) is required for the Ub conjugating activity of UbcD1 (Pan et al., 2017). Using the MARCM system, we found that reduction of Cut expression in *UbcD1* ^*mer1*^ homozygous mutant cells was rescued by over-expression of UbcD1^WT^ (Figure 7A), but not the “catalytic dead” form UbcD1^C85A^ (Figure 7B). Similarly, only UbcD1^WT^ (Figure 7C) but not UbcD1^C85A^ (Figure 7D) was capable of restoring Cut expression in *UbcD1* RNAi cells. Expression of another Notch target, Wg, was also rescued by UbcD1^WT^ (Figures 7E,F) but not UbcD1^C85A^ (Figure 7G) in *UbcD1* RNAi cells. These results demonstrate that the Ub conjugating activity is essential for UbcD1 to ensure Notch activation.

## Discussion

Formation of wings made insects the first group of animals that gained the ability to fly during evolution (Shimmi et al., 2014). The shape, size and venation patterns of insect wings are highly stereotyped and species specific, and these traits are widely used in biology researches ranging from species identification, organ development to evolutionary modelling (Parchem et al., 2007). Our knowledge of the genetic and molecular basis of insect wing development largely originates from studies in the model insect, *Drosophila melanogaster* (De Celis and Diaz-Benjumea, 2003). The *Notch* mutant likely represents one of the first recorded *Drosophila* mutations that affect wing development. Later studies demonstrate that the Notch signal pathway is highly conserved among the insects and regulates various developmental processes across different species. Notch signaling regulates wing margin formation in *Drosophila hydei* (Van Breugel and Langhout, 1983) and sheep blowfly (Davies et al., 1996; Chen et al., 1998), wing morphogenesis in silkworm (Sato et al., 2008; Ling et al., 2015) and pigment patterns in the butterfly wing (Reed, 2004; Reed and Serfas, 2004). Further studies indicate that Notch signaling is required for oogenesis in *Blattella germanica* (Irles et al., 2016) and locust (Song et al., 2019), reproductive constraint in the adult worker honeybee (Duncan et al., 2016), appendage development in silk worm (Liu, 2012) and camouflage patterns in caterpillars (Jin et al., 2020). Recent studies reveal crucial role of Notch signaling during body segmentation in insect species such as cockroaches (Pueyo et al., 2008; Chesebro et al., 2013) and silkworm (Liu, 2013). Whether Notch signaling regulates segmentation in cricket is still under debate (Kainz et al., 2011; Mito et al., 2011), but segmentation in *Drosophila* (Liao and Oates, 2017) and grasshopper (Dearden and Akam, 2000) is likely independent of Notch signaling. These studies highlight the important and diverse roles of Notch signaling, identification of new factors involved in Notch signal transduction will help us to better understand how it operates to control insect development.

Our data presented here suggests a novel role for UbcD1 as a positive regulator of the Notch signaling pathway during fly wing development. Previous studies have found that UbcD1 genetically interacts with the DUB Faf (Cadavid et al., 2000) and E3 ligase Neur (Lai et al., 2001), both of which regulate Dl endocytic trafficking during fly eye development. However, whether and how Dl protein and Notch signaling are affected in *UbcD1* mutant eye disc cells have not been investigated (Cadavid et al., 2000; Lai et al., 2001). Furthermore, Faf is dispensable for fly wing development (Fischer-Vize et al., 1992). The E3 ligase Neur is essential for sensory precursors specification but not wing margin formation and other Notch signaling dependent processes during wing development (Yeh et al., 2000; Lai and Rubin, 2001). Therefore, UbcD1 is likely involved in Notch signaling regulation in multiple tissues and developmental contexts, targeting distinct signal molecules and transduction steps. Our genetic analysis suggests that UbcD1 functions presumably at early steps before the cleavage of full-length Notch protein, but also impacts later transduction events such as Notch trafficking and distribution in the developing wing. The molecular targets of UbcD1 and the exact mechanisms that how UbcD1 impacts Notch signaling still remains elusive.

Alternatively, UbcD1 might regulate Notch signaling indirectly through cellular processes such as endolysosomal trafficking. Our results indicate that UbcD1 is likely required for maturation of late endosomes and following steps towards lysosomal degradation. A crucial event during endosome maturation is Rab conversion, during which the early organizer Rab5 is replaced by the late organizer Rab7. Recent work identifies Dmon1, a member of the Sand1/Mon1 protein family, as a crucial factor for Rab conversion during fly wing development (Yousefian et al., 2013). In fly wing disc cells, LOF of *Dmon1* results in reduced association of Rab7 with endosomes, enhancement of endolysosomal acidification and accumulation of Notch proteins (Yousefian et al., 2013). The high similarity of these LOF phenotypes indicate that UbcD1 might be involved in Rab conversion. The exact role of UbcD1 in the endolysosomal machinery remains an open question.

Given the broad cellular activities of UbcD1, it is not surprising to find that UbcD1 might regulate multiple signaling pathways during wing development. It has been shown that UbcD1 negatively regulates Hh signaling activation in the wing (Pan et al., 2017). When *UbcD1* expression was inhibited by RNAi, down-regulation of Wg was observed in cells located at the D/V boundary due to disruption of Notch signaling transduction. In contrast, accumulation of Wg were found in *UbcD1* RNAi cells at the edge of wing pouch (Figure 7E). The expression of Wg is regulated by signaling pathways other than Notch at this region. For example, in response to cell apoptosis, another cellular event that involves UbcD1 (Ryoo et al., 2002), the JNK pathway is sufficient to induce Wg expression in these cells (Ryoo et al., 2004). Whether and how UbcD1 is involved in these pathways during wing development awaits further investigation.

## Data Availability

The raw data supporting the conclusion of this article will be made available by the authors, without undue reservation.
