# Supplementary figures and images for: The Ubiquitin Conjugating Enzyme UbcD1 is Required for Notch Signaling Activation During Drosophila Wing Development
Source: Front Genet. 2021 Oct 12;12:770853. doi: 10.3389/fgene.2021.770853 (PMC8546230; doi:10.3389/fgene.2021.770853)

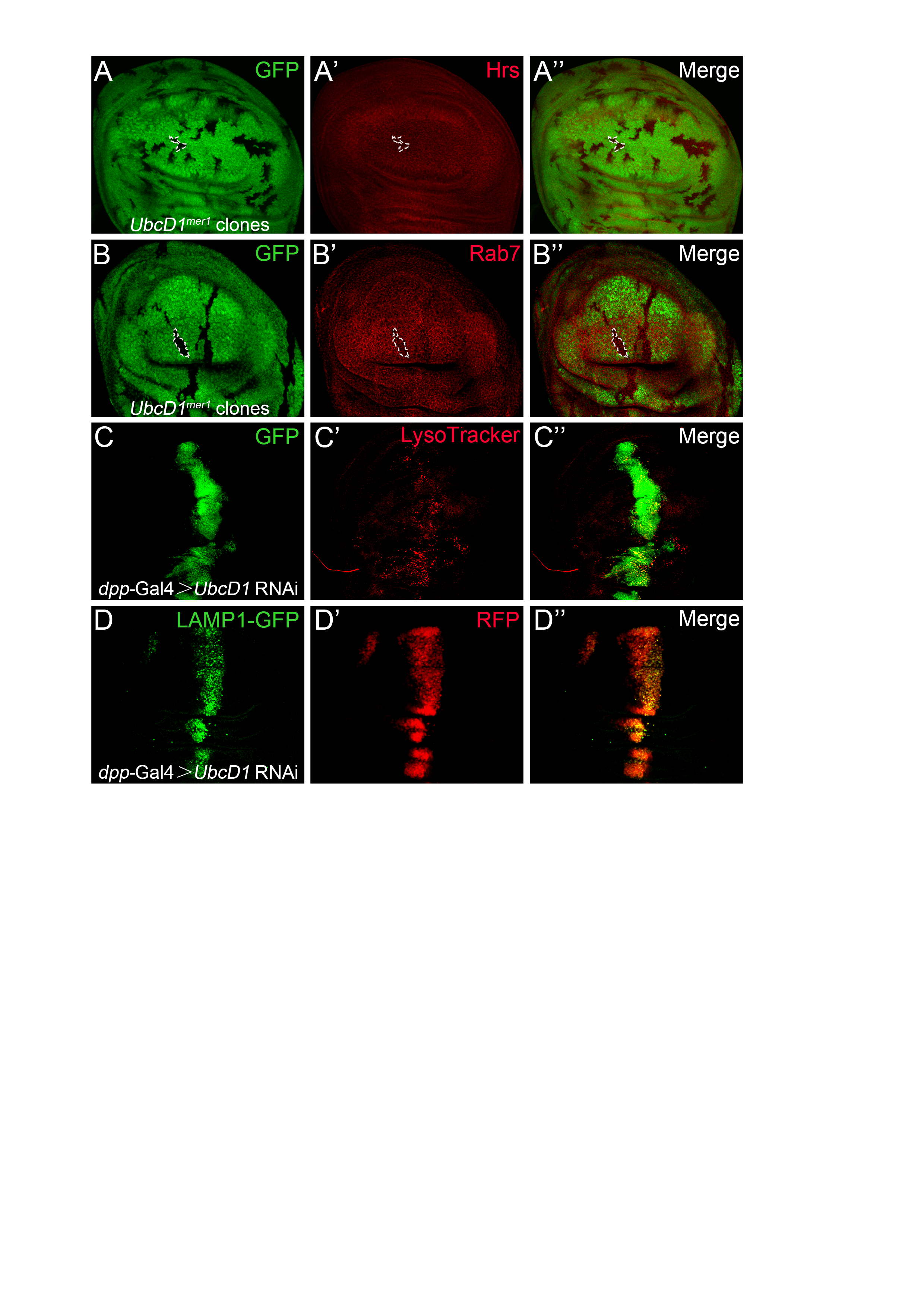

Supplement: Supplementary file 1 [file Image3.TIF]

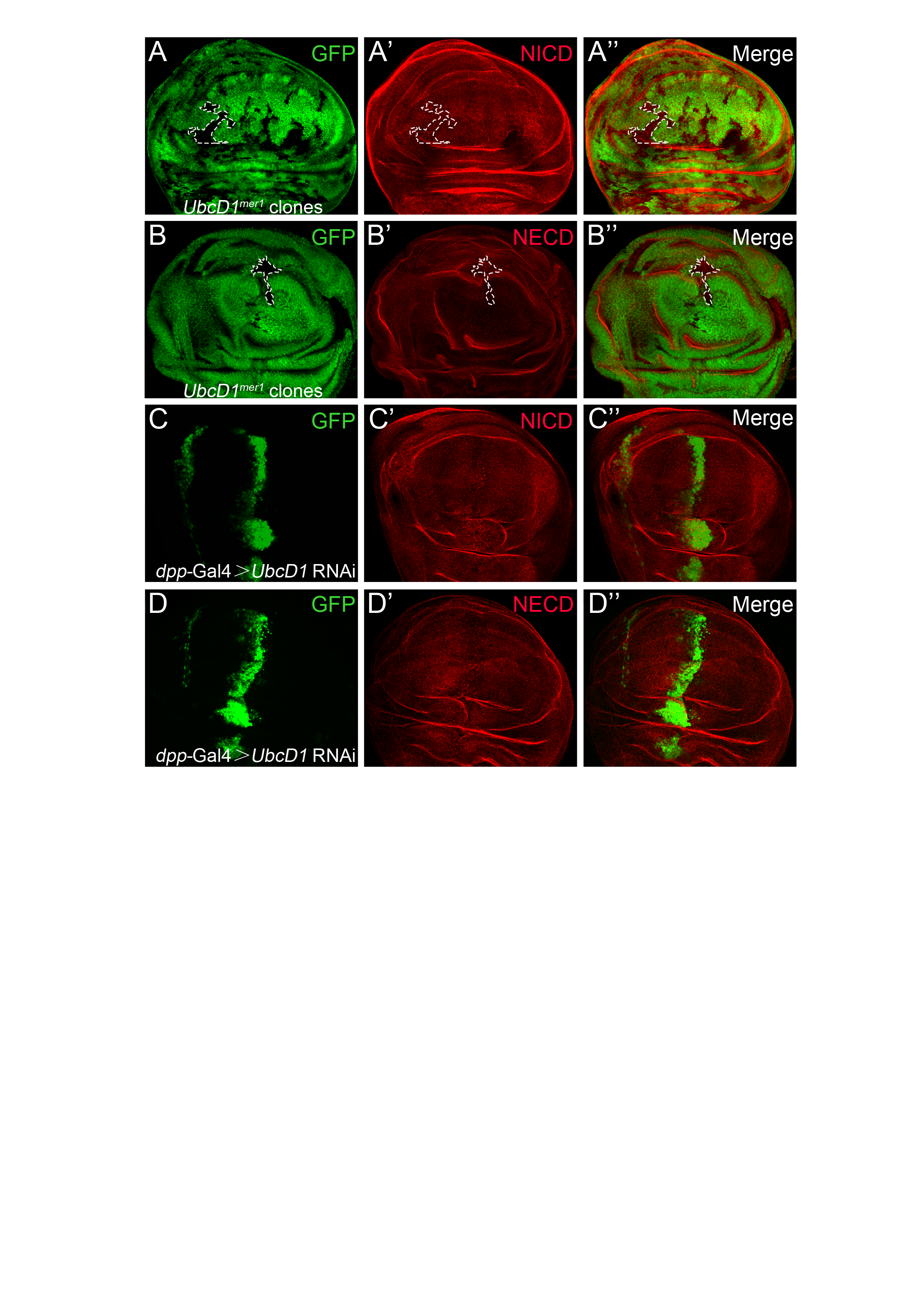

Supplement: Supplementary file 2 [file Image2.TIF]

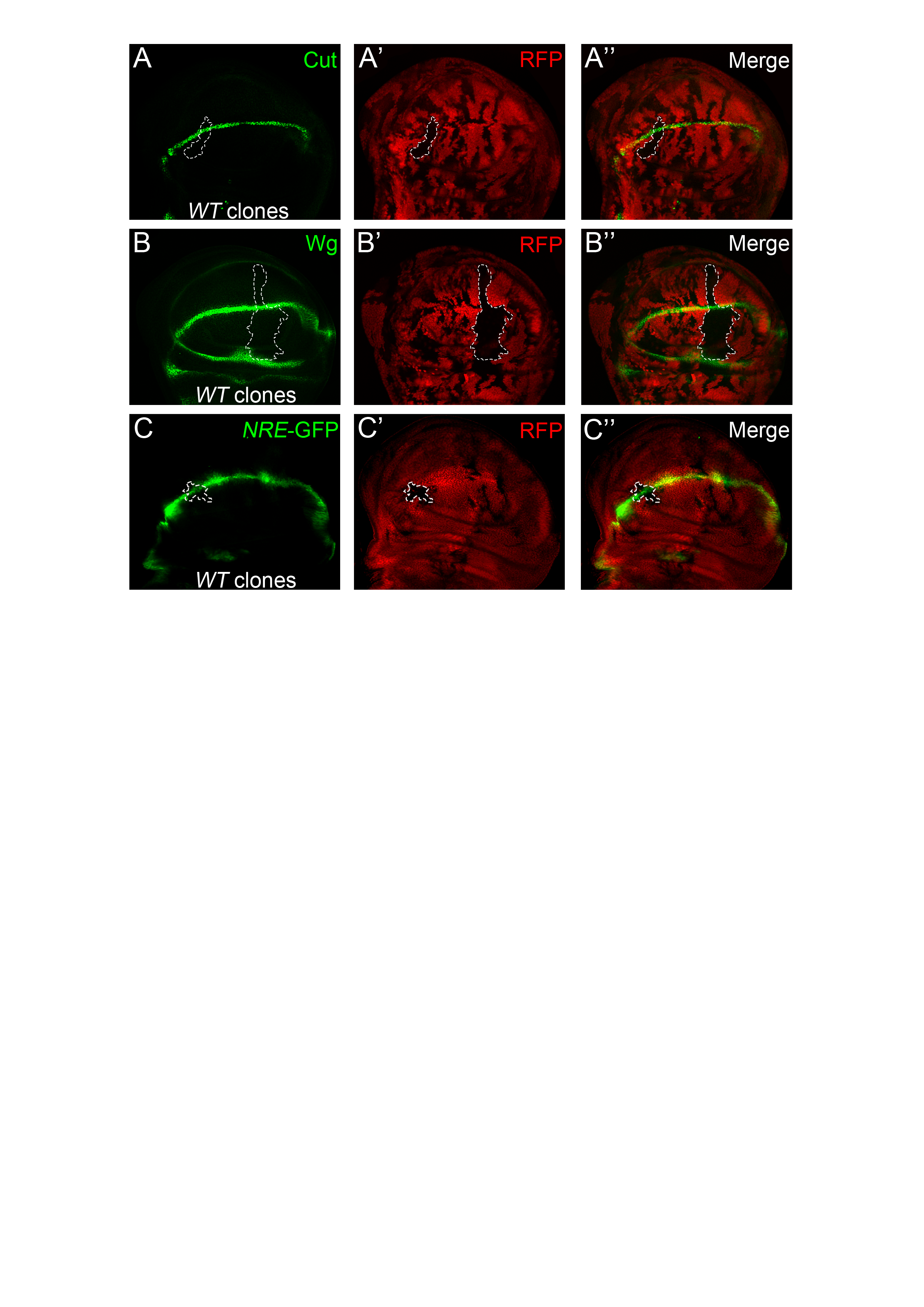

Supplement: Supplementary file 3 [file Image1.TIF]
