# Supplementary material for: The Ubiquitin Conjugating Enzyme UbcD1 is Required for Notch Signaling Activation During Drosophila Wing Development
Source: Front Genet. 2021 Oct 12;12:770853. doi: 10.3389/fgene.2021.770853 (PMC8546230; doi:10.3389/fgene.2021.770853)
Supplement: Supplementary file 4 [file DataSheet1.docx]

**SUPPLEMENTARY FIGURE LEGENDS**

SUPPLEMENTARY FIGURE S1 Expression pattern of Notch signaling targets.

(A-C) In wild type wing discs, the Notch signaling targets Cut (A), Wg (B) and the reporter *NRE*-GFP (C) are expressed by cells at the D/V boundary.

SUPPLEMENTARY FIGURE S2 UbcD1 regulates Notch protein distribution.

(A-B) Immunostaining using both NICD (A) and NECD (B) antibodies show accumulation of Notch proteins in *UbcD1^mer1^* mutant cells. Mutant clones are marked by absence of GFP. Representative mutant clones are circled by dashed lines. (C-D) Immunostaining using both NICD (C) and NECD (D) antibodies show accumulation of Notch proteins in *UbcD1* RNAi cells. The RNAi expressing cells are marked by GFP.

SUPPLEMENTARY FIGURE S3 UbcD1 regulates endolysosomal machinery.

(A-B) Hrs positive early endosomes are unaffected in *UbcD1^mer1^* mutant cells (A). Formation of Rab7 positive endosomes are reduced in *UbcD1^mer1^* mutant cells (B). Mutant clones are marked by absence of GFP. Representative mutant clones are circled by dashed lines. (C-D) Accumulation of LysoTracker (C) and GFP-LAMP1 (D) are found in *UbcD1* RNAi cells. The RNAi expressing cells are marked by GFP (C) or RFP (D).
